# Supplementary material for: Multi‐dataset identification of innovative feature genes and molecular mechanisms in keratoconus
Source: J Cell Mol Med. 2024 Sep 19;28(18):e70079. doi: 10.1111/jcmm.70079 (PMC11412914; doi:10.1111/jcmm.70079)
Supplement: Supplementary file 2 — Material S2. [file JCMM-28-e70079-s002.zip › 7.GSEA analysis/3.DBNDD1/KEGG_PORPHYRIN_AND_CHLOROPHYLL_METABOLISM.html]

Details for gene set KEGG\_PORPHYRIN\_AND\_CHLOROPHYLL\_METABOLISM[GSEA]

|  || Dataset | DBNDD1.group.cls#h\_versus\_l |
| Phenotype | group.cls#h\_versus\_l |
| Upregulated in class | h |
| GeneSet | KEGG\_PORPHYRIN\_AND\_CHLOROPHYLL\_METABOLISM |
| Enrichment Score (ES) | 0.43175957 |
| Normalized Enrichment Score (NES) | 1.392675 |
| Nominal p-value | 0.08196721 |
| FDR q-value | 0.90321386 |
| FWER p-Value | 0.897 |
Table: GSEA Results Summary

  

Fig 1: Enrichment plot: KEGG\_PORPHYRIN\_AND\_CHLOROPHYLL\_METABOLISM      
 Profile of the Running ES Score & Positions of GeneSet Members on the Rank Ordered List

  

| SYMBOL | TITLE | RANK IN GENE LIST | RANK METRIC SCORE | RUNNING ES | CORE ENRICHMENT || 1 | UGT1A8 | na | 313 | 0.502 | 0.1603 | Yes |
| 2 | UGT2B11 | na | 1393 | 0.345 | 0.2456 | Yes |
| 3 | UGT2B4 | na | 2640 | 0.266 | 0.2994 | Yes |
| 4 | UGT1A6 | na | 2660 | 0.265 | 0.3882 | Yes |
| 5 | ALAD | na | 5807 | 0.155 | 0.3499 | Yes |
| 6 | UGT2B28 | na | 6027 | 0.150 | 0.3941 | Yes |
| 7 | MMAB | na | 6362 | 0.140 | 0.4318 | Yes |
| 8 | ALAS2 | na | 9283 | 0.081 | 0.3750 | No |
| 9 | EARS2 | na | 9963 | 0.072 | 0.3797 | No |
| 10 | CPOX | na | 11117 | 0.057 | 0.3656 | No |
| 11 | UROS | na | 11545 | 0.052 | 0.3708 | No |
| 12 | COX15 | na | 11823 | 0.049 | 0.3792 | No |
| 13 | UGT2A3 | na | 13213 | 0.036 | 0.3515 | No |
| 14 | FECH | na | 14399 | 0.027 | 0.3265 | No |
| 15 | UROD | na | 15289 | 0.021 | 0.3078 | No |
| 16 | HMOX2 | na | 16311 | 0.014 | 0.2831 | No |
| 17 | BLVRB | na | 17782 | 0.004 | 0.2422 | No |
| 18 | UGT2B10 | na | 17872 | 0.004 | 0.2408 | No |
| 19 | FTH1 | na | 18016 | 0.003 | 0.2376 | No |
| 20 | HMBS | na | 18457 | -0.000 | 0.2250 | No |
| 21 | PPOX | na | 18874 | -0.003 | 0.2142 | No |
| 22 | UGT2B15 | na | 18962 | -0.004 | 0.2132 | No |
| 23 | FTMT | na | 19994 | -0.013 | 0.1878 | No |
| 24 | GUSB | na | 20109 | -0.014 | 0.1892 | No |
| 25 | COX10 | na | 22102 | -0.033 | 0.1430 | No |
| 26 | HMOX1 | na | 22858 | -0.041 | 0.1351 | No |
| 27 | BLVRA | na | 23513 | -0.050 | 0.1331 | No |
| 28 | HCCS | na | 24333 | -0.061 | 0.1300 | No |
| 29 | UGT2B7 | na | 24690 | -0.066 | 0.1421 | No |
| 30 | ALAS1 | na | 24880 | -0.069 | 0.1599 | No |
| 31 | UGT2A1 | na | 28049 | -0.132 | 0.1132 | No |
| 32 | CP | na | 31366 | -0.235 | 0.0969 | No |
Table: GSEA details [plain text format]

  

Fig 2: KEGG\_PORPHYRIN\_AND\_CHLOROPHYLL\_METABOLISM      
 Blue-Pink O' Gram in the Space of the Analyzed GeneSet

  

Fig 3: KEGG\_PORPHYRIN\_AND\_CHLOROPHYLL\_METABOLISM: Random ES distribution      
 Gene set null distribution of ES for **KEGG\_PORPHYRIN\_AND\_CHLOROPHYLL\_METABOLISM**
